# A New Breast Cancer Discovery Strategy: A Combined Outlier Rejection Technique and an Ensemble Classification Method

**DOI:** 10.3390/bioengineering11111148

**Published:** 2024-11-15

**Authors:** Shereen H. Ali, Mohamed Shehata

**Affiliations:** 1 Communications & Electronics Engineering Department, Delta Higher Institute for Engineering & Technology, Mansoura 35511, Egypt; sherein_h@dhiet.edu.eg; 2 Department of Bioengineering, Speed School of Engineering, University of Louisville, Louisville, KY 40292, USA

**Keywords:** breast cancer, data mining, feature selection, outlier rejection, Harris hawk optimization, ensemble classification

## Abstract

Annually, many people worldwide lose their lives due to breast cancer, making it one of the most prevalent cancers in the world. Since the disease is becoming more common, early detection of breast cancer is essential to avoiding serious complications and possibly death as well. This research provides a novel Breast Cancer Discovery (BCD) strategy to aid patients by providing prompt and sensitive detection of breast cancer. The two primary steps that form the BCD are the Breast Cancer Discovery Step (BCDS) and the Pre-processing Step (P^2^S). In the P^2^S, the needed data are filtered from any non-informative data using three primary operations: data normalization, feature selection, and outlier rejection. Only then does the diagnostic model in the BCDS for precise diagnosis begin to be trained. The primary contribution of this research is the novel outlier rejection technique known as the Combined Outlier Rejection Technique (CORT). CORT is divided into two primary phases: (i) the Quick Rejection Phase (QRP), which is a quick phase utilizing a statistical method, and (ii) the Accurate Rejection Phase (ARP), which is a precise phase using an optimization method. Outliers are rapidly eliminated during the QRP using the standard deviation, and the remaining outliers are thoroughly eliminated during ARP via Binary Harris Hawk Optimization (BHHO). The P^2^S in the BCD strategy indicates that data normalization is a pre-processing approach used to find numeric values in the datasets that fall into a predetermined range. Information Gain (IG) is then used to choose the optimal subset of features, and CORT is used to reject incorrect training data. Furthermore, based on the filtered data from the P^2^S, an Ensemble Classification Method (ECM) is utilized in the BCDS to identify breast cancer patients. This method consists of three classifiers: Naïve Bayes (NB), K-Nearest Neighbors (KNN), and Support Vector Machine (SVM). The Wisconsin Breast Cancer Database (WBCD) dataset, which contains digital images of fine-needle aspiration samples collected from patients’ breast masses, is used herein to compare the BCD strategy against several contemporary strategies. According to the outcomes of the experiment, the suggested method is very competitive. It achieves 0.987 accuracy, 0.013 error, 0.98 recall, 0.984 precision, and a run time of 3 s, outperforming all other methods from the literature.

## 1. Introduction

Breast cancer is a lethal disease that resulted in 10 million fatalities and 19.3 million cases globally in 2020 [1]. With one in every eight women battling breast cancer at some point in their lives, the severity of its effects becomes even more obvious [2]. There are two types of cancerous tumors: malignant and benign. Benign tumors do not cause much harm. However, malignant tumors are aggressive and have the potential to spread to other bodily areas. Breast cancer had a profound effect on women’s lives. Raising awareness and facilitating early diagnosis and prognosis can reduce overall mortality [3]. Early detection of breast cancer can help doctors defeat this illness and improve the prognosis of people with breast cancer [4]. The two most crucial components of the treatment process for breast cancer are early detection and a precise diagnosis made during the disease’s early stages. This can be performed by using artificial intelligence (AI) and data mining (DM) methods during the diagnostic procedure.

AI has emerged as an effective tool in medical imaging and diagnostics in recent decades, exhibiting extraordinary competence in evaluating large medical datasets, such as mammograms and MRIs, frequently obtaining accuracy levels similar to, if not exceeding, human specialists [5,6]. In the domain of breast cancer, AI is useful in early detection and diagnosis, revealing subtle patterns and anomalies that humans may miss [7]. Furthermore, predictive modeling using AI aids in the identification of patient risk factors, allowing physicians to identify individuals who are more likely to develop breast cancer, allowing prompt action and prevention efforts [8].

Nevertheless, a variety of data sources, including computed tomography scans, genomes, mammograms, magnetic resonance imaging, ultrasound, and laboratory results, are needed for effective AI-based disease diagnosis. DM is the process of taking complex datasets and turning them into useful knowledge [9]. The healthcare sector includes a vast quantity of diverse data that can be turned into meaningful information using DM approaches. Then, that valuable information can be used to efficiently assess a variety of ailments. Thus, it is possible to diagnose diseases such as breast cancer more quickly and effectively. This will help to improve the standard of medical care while eliminating waste of medical funds.

The most popular use of DM is classification, which is based on supervised learning techniques [10], whereas ensemble classification is a relatively new classification approach. Afterward, a voting mechanism combines the classifications of the various base classifiers to forecast the class label of the unknown item. The primary principle behind ensemble classification is to take advantage of the base classifiers’ strengths while avoiding their weaknesses. Ensemble classification has been both theoretically and empirically proven to increase classification stability and accuracy compared to a single classifier [11,12].

This study introduces a new Breast Cancer Discovery (BCD) strategy for rapid and precise detection of patients with breast cancer. The BCD consists of two basic steps: the Pre-processing Step (P^2^S) and the Breast Cancer Discovery Step (BCDS). The P^2^S involves three major processes: data normalization, feature selection, and outlier rejection to retrieve appropriate data from inefficient input. Thus, the training of the diagnostic method for accurate evaluation in the BCDS can then be started. The Combined Outlier Rejection Technique (CORT), a revolutionary outlier rejection method, is the main aspect of this study. The two main phases of CORT are the quick rejection phase (QRP), which uses a statistical method, and the accurate rejection phase (ARP), which incorporates an optimization technique. The QRP quickly removes outliers using the standard deviation, and then the ARP removes the rest of the outliers with Binary Harris Hawk Optimization (BHHO). “P^2^S” denotes data normalization to obtain numerical values in datasets that are inside a certain range. Then, Information Gain (IG) is utilized to select the best set of features. Otherwise, CORT is employed to discard inaccurate training data. Additionally, an Ensemble Classification Method (ECM) is employed in the BCDS to recognize breast cancer patients according to the purified data from the P^2^S. The ECM utilizes three classifiers: Support Vector Machine (SVM), Naïve Bayes (NB), and K-Nearest Neighbors (KNN). Considering the outcomes of the experiments, the BCD strategy outperforms other recent strategies as it provides the maximum accuracy, precision, recall, F1-measure, minimum error, and run time.

The major insights of this work can be summarized as follows: (i) A novel strategy known as BCD is employed to accurately diagnose breast cancer patients. (ii) A new outlier rejection strategy known as CORT is offered, which consists of two stages: the QRP and the ARP. (iii) The QRP incorporates a quick rejection approach called standard deviation as a statistical tool. (iv) Subsequently, BHHO is suggested as an optimization method to precisely reject outliers. (v) An ECM is a new blended diagnostic technique used in the BCDS that yields accurate results. (vi) Numerous methods are used to test the efficacy and application of the proposed BCD strategy, including independent evaluations of the suggested CORT and ECM.

The rest of the article is structured as follows: The related work is discussed in Section 2. The suggested research strategy is presented in Section 3. The experimental results are presented in Section 4. A discussion is given in Section 5. Section 6 concludes by discussing the findings and recommendations for the future.

## 2. Related Works

In this section, previous efforts related to breast cancer discovery will be presented.

Silva et al. [13] employed a mixed artificial intelligence system that utilizes fuzzy models and neural network principles to identify breast cancer individuals using fuzzy rules. To demonstrate the practicality of employing fuzzy neural networks, tests were binary classified. The techniques used with the fuzzy neural network included a variety of membership functions. The results revealed that Gaussian membership functions performed best, with 81.4% accuracy, 81.9% sensitivity, and 81% specificity.

Khashei et al. [14] modified the learning process of intelligent models to fit the distinct target functions of classifications. The multilayer perceptron (MLP) classification model was combined with the suggested discrete learning-based approach. Several breast cancer classification datasets were utilized to illustrate the advantage of the proposed discrete learning-based MLP (DIMLP) model. The suggested DIMLP model achieved 94.70% accuracy.

Wei et al.’s [15] use of the Wisconsin breast cancer diagnostic dataset demonstrated the effectiveness of machine learning techniques, specifically the Random Forest technique, in predicting breast cancer cases. The random forest technique yielded 95% accuracy.

Ahmed et al. [16] presented a methodology for classifying breast cancer instances as benign or malignant using a one-dimensional convolutional neural network (1D CNN) for acquiring features and machine learning techniques, particularly extreme gradient boosting (XGBoost). The XGBoost technique presented an accuracy of 98.24%.

Uddin et al. [17] classified breast cancer as benign or malignant lesions via several types of machine learning classifiers. To select the best model, the accuracy of each one was evaluated. The evaluation found that the voting methods had the highest accuracy, at 98.77%.

Singh et al. [18] integrated soft computing methodologies with a variety of machine learning methods to create a breast cancer prediction model. The system’s effectiveness was evaluated using WDBC datasets, and the outcomes showed that the combined approach performed best in BC classification, with an accuracy of 98.96%. Table 1 introduces a comparative review of various breast cancer discovery techniques.

In addition, previous efforts related to outlier rejection in breast cancer discovery will be reviewed. Chomatek et al. [19] provided a set of objectives enabling the accurate detection of outliers using a multi-objective genetic algorithm (MGA).

Yusuf et al. [20] investigated the effects of outliers and feature elimination via seven different machine learning algorithms. Jensch et al. [21] recommended robust sparse ensemble for outlier detection (ROSIE), which incorporates three basic and resilient algorithms for outlier detection and feature selection. Mohamed et al. [22] proposed a combination approach incorporating the CNN design and the Ebola optimization technique to detect breast cancer using gene expression data. Array–Array Intensity Correlation (AAIC) was deployed to eliminate outliers.

Lopes et al. [23] explained the capacity for outlier identification utilizing ensemble hypotheses based on genetic expression and clinical parameters for outlier patients. Table 2 depicts recent approaches to outlier rejection used in assessment strategies.

## 3. The Proposed Breast Cancer Discovery (BCD) Strategy

This section will provide an explanation of the suggested BCD strategy. BCD is a novel discovery or diagnostic approach that uses the BCD dataset, which comprises digital images of a fine-needle aspiration samples collected from patients’ breast masses, to quickly and more accurately identify breast cancer patients.

**Figure 1:**
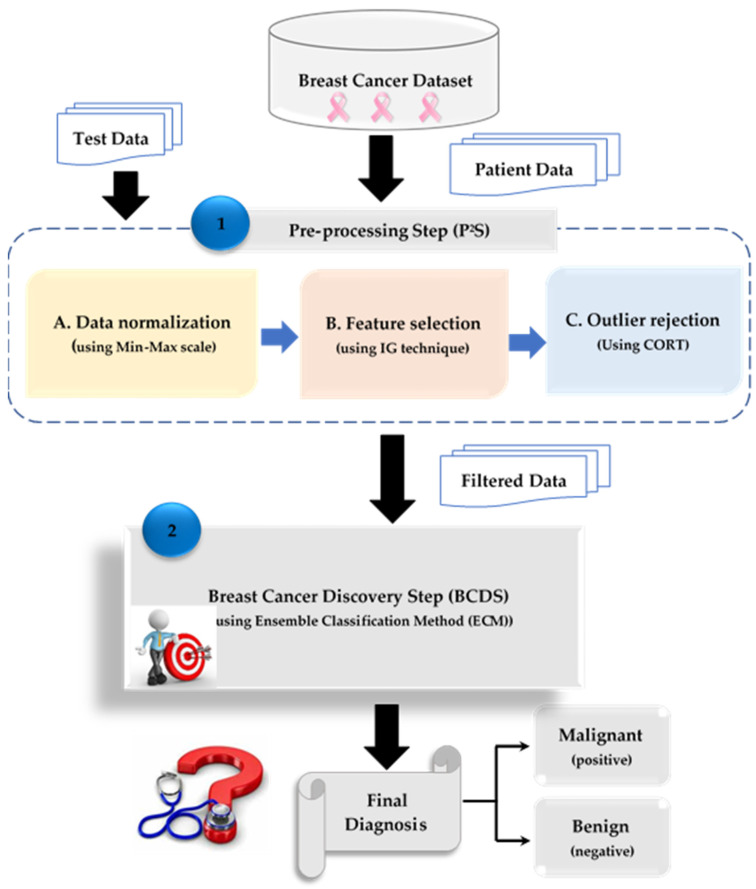
The proposed Breast Cancer Discovery (BCD) Strategy.

The Breast Cancer Discovery Step (BCDS) and Pre-processing Step (P^2^S) make up the BCD strategy, as illustrated in Figure 1. The filtered dataset can be utilized in the BCDS to enable a detection model to produce a quicker and more nuanced diagnosis, even though the employed BCD strategy dataset may be purified and cleared of both insignificant features and outliers in the P^2^S.

Three primary operations, namely, data normalization, feature selection, and outlier rejection, are the foundation of P^2^S data filtering. One preliminary method called data normalization looks for numerical values in datasets that fall into a predetermined range utilizing min–max scaling. Feature selection is intended to eliminate less important aspects, and outlier rejection serves to exclude data whose behavior is truly unusual. In order to give a prompt and sensitive diagnosis, the BCDS diagnostic model can be trained on a legitimate dataset that only contains valid data and the most useful features for breast cancer patients.

Actually, there are two primary categories of feature selection approaches: filter and wrapper. Filter methods are faster than wrapper methods. On the other hand, outlier rejection techniques are divided into three primary classes: statistical, cluster, and neighbor procedures [24,25]. Information gain (IG) acts as a filter mechanism in P^2^S feature selection to rapidly identify the most informative collection of features [26].

Next, in order to produce a legitimate dataset devoid of outliers, a novel outlier rejection method known as the Combined Outlier Rejection Technique (CORT) is applied. In order to precisely adopt the Ensemble Classification Method (ECM) as a diagnostic tool to provide a prompt and sensitive diagnosis, the dataset is finally handed from the P^2^S to the BCDS.

**Figure 2:**
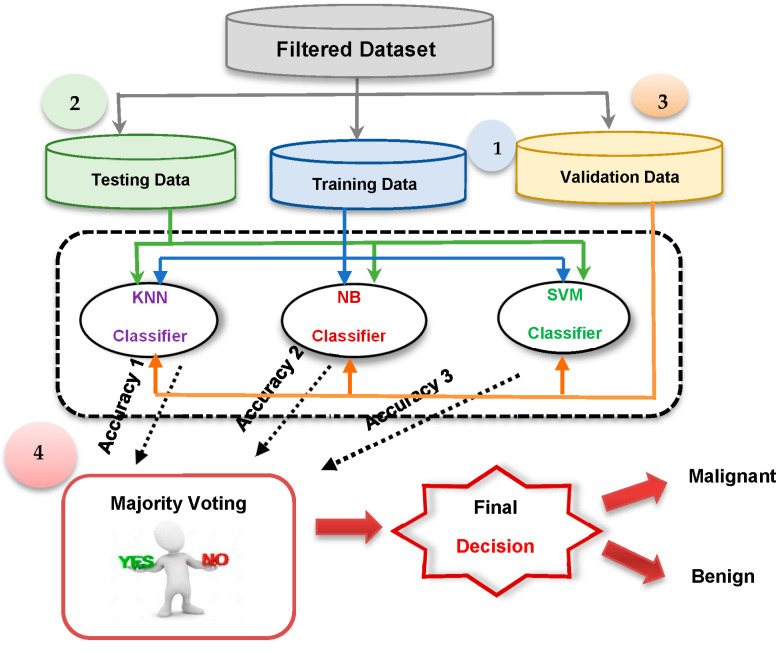
The steps of the Ensemble Classification Method (ECM).

The ECM comprises three primary machine learning diagnostic techniques: NB [27], KNN [28], and SVM [28]. They are integrated using the majority voting approach to make the proper choice, as illustrated in Figure 2. Each of these techniques is trained in parallel using a common training dataset. They are then verified to make decisions independently after being tested concurrently on a common test set. Based on their final choices, majority voting is used to make an accurate determination based on the diagnosis that receives the most votes.

There are four basic phases to adopting the ECM, as shown in Figure 2. The first phase involves training the NB, KNN, and SVM classifiers, and the second phase involves testing them. Following the third phase’s validation of these procedures, majority voting is used to make the ultimate decision based on their recommendations. This is followed by a thorough description of the suggested outlier rejection technique.

### 3.1. The Proposed Combined Outlier Rejection Technique (CORT)

As with non-informative features, outliers (inaccurate data) influence the classifier. Inaccurate data lead the detection model astray from providing a precise determination. Therefore, outliers in a medical dataset cause overfitting, which reduces the efficiency of detection techniques [9]. As a result, removing such incorrect data rather than including them in the dataset is a vital operation that must be undertaken on the dataset gathered from individuals prior to employing the detection technique. Due to the elimination of outliers, detection will then produce precise and rapid results.

Essentially, there are three main kinds of rejection techniques used to remove inaccurate data: statistical, cluster, and neighbor techniques. Cluster approaches view outliers as byproducts, but statistical methods view outliers as data that deviate from a specific distribution [29]. Neighbor approaches compare each training patient’s degree of difference from neighbors via various metrics. These techniques can rapidly rule out anomalies, but they are unable to deliver a reliable set of training data. Recently, optimization techniques have become essential for correctly excluding erroneous data from training datasets [30].

In the P^2^S, CORT is used as a new rejection strategy to exclude inaccurate data before training the diagnostic model for a more reliable diagnosis, as illustrated in Figure 1. CORT is divided into two primary phases: (i) the Quick Rejection Phase (QRP), which includes a statistical approach in the interest of speed, and (ii) the Accurate Rejection Phase (ARP), which employs an optimization technique in the interest of accuracy. The QRP can promptly eliminate many outliers, and the ARP is a viable procedure for eliminating the remaining outliers.

Thus, the steps involved in the outlier rejection technique are as follows: The medical dataset is first submitted to the QRP for fast outlier rejection, and the results of this stage are then submitted as input to the ARP for exact outlier rejection. The standard deviation approach is adopted in the QRP to swiftly eliminate a large number of outliers from the training dataset [31].

After the outliers have been eradicated, the training dataset is sent to an ARP optimization technique to eliminate the remaining inaccurate information. The optimization technique is a Binary Harris Hawk Optimization (BHHO) [32], which was motivated by the “surprise pounce” cooperative behavior of Harris hawks in the wild. It is a recent addition to the category of algorithms based on swarm intelligence [33]. A subset of the training data given from the QRP will be used to perform BHHO within a single iteration of the optimization method execution.

Algorithm 1 illustrates the several sequential stages needed in the creation of CORT. Initially, let us consider a dataset containing “tm” training examples. To swiftly eliminate “out” outliers from this dataset, a standard deviation threshold is applied in the QRP, and the remaining training examples are considered “rest”, where rest =tm−out. Once the remaining outliers have been precisely eliminated via the optimization technique in the ARP, the dataset comprising “rest” training instances is sent to it. Only accurate data containing “ins” training instances, where ins<rest, are provided. During the QRP, each instance that deviates over d times from the mean of its related class is identified via the SD method [31]. SD calculates how far each instance in the training set departs from the class mean. To demonstrate the concept, consider $SD(I_{i},cl)$ to be the standard deviation, which uses (1) to quantify the ith case $\;I_{i}$’s degree of deviation from the mean of the related class cl.

(1) $$SD(I_{i},cl)=|I_{i}-\mu_{cl}\;|*{\sigma_{cl}}^{-1}$$

where the vector of the ith training instance is $\;I_{i}$, the standard deviation vector in the class cl  is ${\sigma_{cl}}^{-1}$, and the mean vector in the class cl is $\mu_{cl}$.

Each instance in the training dataset is identified by ‘b’ features. Therefore, $I_{i}$, ${\sigma_{cl}}^{-1},$ and $\mu_{cl}$ are expressed as three vectors; [$I_{i}$ ($f_{1}$), $I_{i}$ ($f_{2}$),… $I_{i}$ ($f_{b}$)], ${\left[\sigma_{cl}\;(f_{1}),\;\sigma_{cl}\;(f_{2}),\ldots \;\sigma_{cl}\;(f_{b})\right]}^{-1}$, and [$\mu_{cl}$ ($f_{1}$), $\mu_{cl}$ ($f_{2}$), … $\mu_{cl}$ ($f_{b}$)], respectively, where each vector size equals ‘b’, which is the number of features in the dataset. To find the mean $\mu_{cl}$ ($f_{i}$), (2) is used in accordance with the ith feature $f_{j}\;$ in the class cl.

(2) $$\mu_{cl}\left(f_{j}\right)=\frac{\sum_{i=1}^{d}I_{i}\left(f_{j}\right)}{d}$$

where $I_{i}\left(f_{j}\right)$ consists of the training data values $\left[I_{1},I_{2},\ldots .I_{d}\right]$ determined by the ith feature $f_{j}$ in the class cl. There are d training cases in the class cl. Utilizing (3), one may determine the standard deviation ${\sigma_{cl}}^{-1}$ for each feature $f_{j}$ in the class cl.

(3) $$\sigma_{cl}\left(f_{j}\right)=\sqrt{\frac{\sum_{i=1}^{d}{\left[I_{i}\left(f_{j}\right)-\mu_{cl}\left(f_{j}\right)\right]}^{2}}{d-1}}$$

In order to identify and remove the outliers, the SD values are afterwards assessed against trd as a threshold value, where $I_{i}$ is considered an outlier if $SD\;(I_{i},cl)>trd.$ The value of trd is actually an aggregate of each instance’s average deviation from the mean of the relevant class cl, as shown in (4).

(4) $$trd=\sum_{cl=1}^{u}h_{cl}$$

where ${trd}_{cl}$ is the mean variance in class cl and u is the number of classes. It is actually possible to determine $trd_{cl}$ using (5).

(5) $${trd}_{cl}=\frac{\sum_{i=1}^{d}\left\|I_{i}-\mu_{cl}\right\|}{d}$$

Following the application of the SD approach in the QRP, “*tm*” training instances are purged of anomalies to produce “rest” instances, where rest<tm. The dataset comprising “rest” training instances will be imported into the ARP in order to generate the initial population for the BHHO that will be run. Actually, there are four stages of exploitation and two stages of exploration for HHO. In an attempt to improve the caliber of output, it uses a variety of intelligence techniques in a selfish plan.

- Initialization stage

The fitness and search spaces are displayed in the first phase. The startup process, based on a population of hawks, is initiated. Additionally, values are given to all parameters [33].

- Exploration stage

The Harris hawks are evaluated as potential solutions in HHO. Two methodologies are used to calculate the fitness based on the intended prey. This phase is described by (6):

(6) $$x\left(J+1\right)=\left\{\begin{array}{rr} x_{rand}\left(J\right)-r1\left|x_{rand}\left(J\right)-2r2x\left(J\right)\right|, & \;\;p\geq 0.5\\ x_{prey}\left(J\right)-x_{m}\left(J\right)-r3\left(LB+r4\left(UB-LB\right)\right), & \;\;p<0.5\; \end{array}\right.$$

Assume that X(J) represents the hawks’ position and X_prey (J) represents the prey’s position. Next, using Equation (6), the location of the hawks during the exploration phase for cycle number  (J+1) is determined based on the probability p.

A hawk’s position for iteration number (J+1) is determined by taking into account the positions of prey and other hawks when p<0.5. The position for iteration (J+1) is also determined by randomly choosing hawks, indicated by X_rand (J), for p≥0.5.

The lower and upper bounds are indicated by LB and UB, where r1, r2, r3, r4, and p are the random variables. In (7), the mean location is denoted by $X_{m}\left(j\right)$, which is calculated as follows:

(7) $$X_{m}\left(J\right)=\frac{1}{Pop\_size}\sum_{i=1}^{Pop\_size}X_{i}\left(J\right)$$

where Pop_size denotes the population’s size.

- Exploration to exploitation

Depending on the prey’s escape energy G, the HHO transitions from the exploration stage to the exploitation stage. It is represented by (8):

(8) $$G=2G_{0}\left(1-\frac{J}{IT\_max}\right)$$

where IT_max indicates the maximum number of cycles and $G_{0}\;$ indicates the prey’s initial energy, which is randomly selected from the range [−1, 1].

- Exploitation stage

This stage is composed of four steps for the parameter sets, including (1) soft siege, (2) hard siege, (3) soft siege with progressive speedy dives, and (4) hard siege with progressive dives. A summary of these steps is illustrated as follows:

In HHO, the prey’s escape prior to the hawks’ unexpected attack is designed. e is a random number that represents the prey’s prospects of escaping successfully (e<0.5) or unsuccessfully (e ≥ 0.5) prior to the hawks’ unexpected attack.

The hawks will launch a soft or hard siege to catch the prey based on whether the prey manages to flee or not. For |G| ≥ 0.5, a soft siege is expressed by Equations (9)–(11).

(9) $$X\left(J+1\right)=∆X\left(J\right)-G\left|T\times X_{prey\left(J\right)}-X\left(J\right)\right|$$

(10) $$∆X\left(J\right)=X_{prey}\left(J\right)-X\left(J\right)$$

(11) $$T=2\left(1-rand\right)$$

where *T* indicates the power of the prey’s random jump.

Similarly, Equation (12) represents a hard siege, which occurs for |G|<0.5.

(12) X(J+1)=X(J)−G|∆X(J)|

The HHO method operates for |G|≥0 and e<0.5 and depicts the prey’s leapfrog movement for a progressive fast dive using the Levy flight concept. Hawks’ successful or unsuccessful dive is calculated using Equation (13).

(13) $$Y=X_{prey}\left(J\right)-G\left|T\times X_{prey}\left(J\right)-X\left(J\right)\right|$$

Hawks use Equation (14) to take a dive based on Levy flight in the event that the dive in Equation (13) is unsuccessful.

(14) $$Z=Y+Q\times Levy\left(dim\right)$$

where *Q* is a random variable and dim is the dimension of the problem. The function of levitation [32] in Levy’s calculation method is as follows:

(15) $$Levy\left(x\right)=0.01\times \frac{u\times \sigma }{{\left|\upsilon \right|}^{\frac{1}{\beta }}}$$

(16) $$\sigma ={\left(\frac{\Gamma \left(1+\beta \right)\times \sin\left(\frac{\pi \beta }{2}\right)}{\Gamma \left(\frac{1+\beta }{2}\right)\times \beta \times 2^{\frac{\beta -1}{2}}}\right)}^{\frac{1}{\beta }}$$

Here, β is a constant with a value of 1.5, and u and v are random variables with values in the range [0, 1]. Therefore, using Equations (13) and (14) as a guide, the final process for adjusting the hawks’ location during this dive is calculated as follows:

(17) $$X\left(J+1\right)=\left\{\begin{array}{c} Y\;\;\;\;\;\;if\;F\left(Y\right)<F\left(X\left(J\right)\right)\\ Z\;\;\;\;\;if\;F\left(Z\right)<F\left(X\left(J\right)\right) \end{array}\right.$$

Here, F represents the problem’s fitness function.

In a similar manner, Equation (17) calculates the hawks’ position during the hard siege stage with successive dives, which happens for |G|<0.5 and e<0.5.

Here, Y and Z are given by Equations (18) and (19).

(18) $$Y=X_{prey}-G\left|T\times X_{prey}-X_{e}\left(J\right)\right|$$

(19) $$Z=W+Q\times Levy\left(dim\right)$$

The population of Harris hawks is initialized by the HHO algorithm. The fitness function of the potential solution is then calculated. A KNN classifier is used to assess the fitness value. The repetition of the preceding procedures will continue until the halting condition is met. In general, continuous optimization concerns are intended to be handled by HHO. However, binary HHO ought to be applied to binary optimization tasks such as feature selection methods.

Therefore, a binary element, either zero or one, is the only solution. As a representation of the solution, a one-dimensional vector with a length equal to the number of features is used. HHO was created to address challenges involving continuous optimization. For challenges involving continuous optimization, HHO was created. It is not possible to address the feature selection issue by merely running binary HHO. Transfer functions are those that create a binary representation of the real-valued search agent. This study presents a binary HHO by combining the modified rule in Equation (20) with the S-shaped (sigmoid) transfer function according to Equation (21).

(20) $$X\left(x\right)=\frac{1}{1+e^{{-}^{x}}}$$

(21) $$x_{binary=\left\{\begin{array}{c} 1\;\;\;\;\;\;\;\;\;if\;rand<X\left(x\right)\\ 0\;\;\;\;\;\;\;if\;rand\;\geq X\left(x\right) \end{array}\right.\;}$$

Here, rand ∈ [0, 1] is a random number, and X(x) are the binary forms of the solution obtained through Equation (21). A KNN will be employed as a fitness (or assessment) method to find the most suitable solution in order to implement BHHO in the ARP [32].

Next, for the specific feature selection produced via KNN, the fitness function is utilized to assess each unique solution so as to strike an equilibrium between the highest classification accuracy and the least number of selected features. A fitness function is described as follows to take each one into account:

(22) $$Fitness=\alpha \;\gamma_{R}\left(D\right)+\delta \frac{\left|R\right|}{\left|N\right|}$$

Here, $\gamma_{R\;}\left(D\right)\;$ represents the KNN’s degree of classification error. Additionally, | N | is the total number of features in data sets, and | R | is the number of chosen variants of features. α∈ [0, 1] and δ=(1−α) are two parameters relating to the importance of classification accuracy and subset length, as suggested in [34], where *α* = 0.99 and *β* = 0.01.
**Algorithm 1: Combined Outlier Rejection Technique (CORT)****Input:** TR=(tm,IF); input training dataset of ‘$d_{i}$’ items expressed by $DD=\left\{I_{1},I_{2},\ldots I_{d}\right\}$ in which each item $I_{i}\in rest$ is expressed as an ordered set of ‘b’ features; $I_{i}\left(f_{1},f_{2},\ldots f_{b}\right)=\left[f_{i1},f_{i2},\ldots f_{ib}\right]$, TE=(Q,IF); Testing dataset. Input target classes expressed by the set $TC=\left\{benign,malignant\right\},\;\;t$: Maximum iterations, $p_{op\_size}$: population size; **Output:** Optimal Solution **Steps:** //Implement Quick Rejection Phase (QRP)// 1. eliminate ‘out’ outliers using standard deviation method to obtain ‘rest’ valid training data, rest=tm−out. //Implement Accurate Rejection Phase (ARP)// 2. Generate the initial position for hawks X$\left(i=1,2,3,\ldots \ldots {Pop}_{{\_}_{size}}\right)$ 3. Evaluate the fitness of initial position 4. Set $X_{prey}$ = fitness value of X 5. for j=1 to t 6.       $for\;i=1\;to\;size\left(X_{i}\right)\;do$ 7.                Update escape energy G via (3) 8.                  if (|G| ≥ 1) then 9.                          Update the position via (1) 10.                    Determine the likelihood via sigmoid transfer function 11.                      Update new position of the hawk using (21) 12.                  if (|G| < 1) then 13.                      if (|G| ≥ 0.5 and e≥0.5) then 14.                              Update position via (4) 15.                              Determine the likelihood via sigmoid transfer function 16.                                Update new position of the hawk using (21) 17.                      else if (|G| < 0.5 and e ≥ 0.5) then 18.                              Update position via (7) 19.                              Determine the likelihood via sigmoid transfer function 20.                                Update new position of the hawk using (21) 21.                      else if (|G| ≥ 0.5 and e<0.5) then 22.                              Update position via (12) 23.                              Determine the likelihood via sigmoid transfer function 24.                                Update new position of the hawk using (21) 25.                      else if (|G| < 0.5 and e<0.5) then 26.                              Update position via (13) 27.                              Determine the likelihood via sigmoid transfer function 28.                                Update new position of the hawk using (21) 29.                      Compute the fitness of the updated population and then update the value $X_{prey}$ 30.          End for 31.   End t 32. Select the optimal solution

### 3.2. Breast Cancer Discovery Step (BCDS)

The actual breast cancer detection takes place in the BCDS, where three different types of base classifiers, constituting an ensemble, are trained using a subset of the available dataset. The employed base classifiers are (a) SVM [27], (b) NB [28], and (c) KNN [28]. A new unlabeled item can be classified by collecting the classification decisions from the three base classifiers. Then, the final breast cancer decision with more votes wins. According to Figure 2, there are four main steps for implementing the ECM model. NB, KNN, and SVM are trained in the first step and then tested in the second step. These techniques are validated in the third step, and finally, an MV is conducted to give the final decision.

## 4. Experimental Results

This section will describe the accomplishment of the proposed BCD strategy for early breast cancer diagnosis. The strategy consists of two steps, namely, the P^2^S and BCDS. The BCD strategy is executed in a sequence of phases. To recognize numeric values in the datasets within a specific range, the P^2^S initially employs the data normalization technique. Next, the IG method is applied to choose the optimal set of features. Lastly, the proposed outlier rejection method, known as CORT, is put in place to remove ineffective training instances from the dataset for exploring breast cancer. In order to detect breast cancer, the filtered data are ultimately transmitted from the P^2^S to the ECM in the BCDS.

The suggested strategy is implemented in two essential cases. In the initial case, after using IG as a feature selection technique, the promoted CORT approach is applied to the breast cancer dataset and examined with other relevant outlier rejection approaches. In the second case, the recommended BCD strategy is then evaluated with other alternatives. Our implementation relies on a dataset of breast cancer patients that includes digital representations of samples collected for fine-needle aspiration (FNA) cytology (i.e., biopsy—the gold standard technique) from the patients’ breast masses [35,36]. Patients classified as benign do not have breast cancer, whereas patients classified as malignant have the disease. The effectiveness of the approaches used is evaluated using a confusion matrix (i.e., a table that specifies the achievement of a classification algorithm) metrics such as accuracy, error, precision, recall, and F1-measure [28,31]. Therefore, various equations are employed to describe the confusion matrix, as shown in Table 3, where TP, TN, FP, and FN represent the counts of true positive, true negative, false positive, and false negative outputs, respectively. Five-fold cross-validation is applied, splitting the breast cancer dataset into five equal partitions. While 20% (one partition) of the data are used as a test set, the other 80% are used as training sets.

### 4.1. Description of the BCD Dataset

The BCD strategy’s efficacy in identifying breast cancer tumors by utilizing every possible feature was assessed via the WBCD dataset. Datasets developed by Dr. William Wolberg were received from hospitals affiliated with the University of Wisconsin in the United States. The UCI Machine Learning Repository has the WBCD dataset available online [35]. The WBCD dataset contains 699 samples (one per patient) acquired by fine-needle aspiration of human breast tissues, including 458 (65.5%) from patients with benign breast tumors and 241 (34.5%) from patients with malignant breast tumors. Nine features were evaluated in each sample, and the values obtained were expressed using an integer value scale of ten points, with one representing a normal state and ten representing the most abnormal state. The features include clump thickness, uniformity of cell size, uniformity of cell shape, marginal adhesion, single epithelial cell size, bare nuclei, bland chromatin, normal nucleoli, and mitoses. Each sample is assigned a class name (benign or malignant). In total, 16 of the 699 samples have missing values for the ‘Bare Nuclei’ feature. Eliminating those 16 instances is the preferred method, in accordance with other cutting-edge studies. Out of 683 full samples, 339 (35%) are malignant and 444 (65%) are benign.

The image pre-processing and analysis procedures were as follows: (1) the cellular nuclei of the FNA slide were illuminated with a microscope; (2) a digital camera and a frame-grabber board were utilized to scan the well-identified slide piece extracted from the FNA sample; (3) Xcyt software (version 1.0)was applied to separate each of the nuclei interactively, with a computer mouse indicating the estimated boundary of the nucleus (following that, the exact boundaries were automatically recognized) [37].

### 4.2. Testing the Combined Outlier Rejection Technique (CORT)

This part will demonstrate the effectiveness of CORT as a novel outlier rejection technique by putting it to the test against other current rejection methods and demonstrating how well it rejects invalid training data. Multi-objective Genetic Algorithm (MGA) [19], Outliers Correlation Feature Selection (OCFS) [20], Robust Sparse ensemble for outlier detection and feature selection (ROSIE) [21], Array–Array Intensity Correlation (AAIC) [22], and Ensemble Outlier Detection Approach (EODA) [23] represent several of the existing outlier rejection techniques, as shown in Table 2. To test these techniques and show how effective CORT is in comparison to alternative approaches, the KNN classifier is employed as the standard classifier using the same input data to avoid any bias.

The accuracy, error, precision, recall, F1-measure, and run time of the employed outlier rejection approaches are shown in Table 4. In fact, CORT defeated other outlier rejection techniques by providing the highest performance results.

CORT achieved an accuracy of 95%, indicating a high degree of correct classifications. Additionally, the recall of 94.2% suggests that CORT successfully identified a substantial portion of positive cases. The F1-measure of 94.29% and precision of 94.4% demonstrate a strong balance between precision and recall. Furthermore, the run time of 5 s indicates the speedy execution of CORT. Lastly, the error of 5% indicates a low rate of incorrect classification.

### 4.3. Testing the Breast Cancer Discovery (BCD) Strategy

This section will compare the suggested BCD strategy to the other diagnostic strategies discussed in [13,14,15,16,17,18]. Actually, there are numerous phases involved in implementing the BCD strategy; the first one is data normalization, which is a pre-processing technique used to identify numerical values in datasets that fall into a predefined range. After that, the WBCD dataset’s most important features are chosen using the feature selection approach. Following that, CORT is used to discard training data that are not valid. In order to provide an accurate diagnosis rapidly, ECM is ultimately trained using the adjusted data.

The accuracy, error, precision, recall, F1-measure, and execution time of the employed diagnostic strategy are displayed in Table 5.

The evaluation results showed that the proposed BCD strategy outperformed the other techniques. The BCD strategy achieved an accuracy of 98.7% and error of 1.3%, indicating a high rate of correct classifications. Additionally, the recall of 98% suggests that the BCD strategy successfully identified a substantial portion of positive cases. The F1-measure of 98.19% and precision of 98.4% demonstrate a strong balance between precision and recall. Furthermore, the run time of 3 s indicates the speedy execution of the BCD strategy.

This can be justified in part by the fact that the BCD strategy relies on employing a pre-processing step that comprises features selection and outlier rejection methods to filter the WBCD dataset [35,36] before performing the BCDS (i.e., the final diagnostic classification step) using an ECM; it is therefore faster than state-of-the-art techniques in [13,14,15,16,17,18].

According to these findings, the BCD strategy has a wide range of benefits and some drawbacks. While the BCD strategy is a scalable, precise, rapid, and efficient technique, it also uses single-label data, has a high degree of complexity, and works with small datasets. An ECM, as a diagnostic procedure, can make decisions based only on reliable, noise-free data, which makes the BCD strategy a quick diagnostic strategy. Its ability to diagnose patients by integrating a variety of features makes it an effective model. After dropping outliers and irrelevant features from the data, the BCD strategy may precisely diagnose breast cancer patients utilizing an ECM. BCD is a useful strategy because it can diagnose patients quickly and precisely. In order to precisely identify patients, the BCD strategy can handle a vast number of features and choose the most appropriate set of them prior to applying an ECM.

While the BCD strategy uses the 683 instances from the WBCD dataset [35,36] to illustrate different contemporary techniques, additional cases should be evaluated in order to yield more precise conclusions. BCD is a vertical classification that divides instances into benign and malignant groups. As a result, multi-label data in the dataset should be utilized to evaluate BCD strategy. Since BCD is a two-layer strategy with multiple phases that are implemented sequentially in each layer, it is a complex model. Table 6 enumerates the benefits and drawbacks of the BCD strategy.

## 5. Discussion

Researchers are interested in finding suitable therapy as soon as possible for breast cancer patients who are identified in a timely and precise way. In actuality, breast cancer patients are not properly diagnosed with the current diagnostic techniques. Therefore, in contrast to many other recent diagnostic procedures, this research focused on presenting a new strategy termed BCD that might provide a quick and reliable diagnosis of breast cancer patients.

Two situations involving the application of the BCD strategy have yielded experimental results: the first involves testing CORT against alternative outlier rejection procedures, and the second involves testing the BCD strategy against alternative diagnostic tactics. A total of 683 examples, of which 80% were used for training and 20% for testing using five-fold cross-validation, comprised the WBCD dataset [35,36], which was used for both situations.

As per the initial situation, the CORT approach revealed better results than other techniques such as MGA [19], OCFS [20], ROSIE [21], AAIC [22], and EODA [23]. KNN was used as a typical classifier to gauge how well these rejection techniques performed.

Regarding accuracy, CORT achieved 95%, while MGA [19], OCFS [20], ROSIE [21], AAIC [22], and EODA [23] presented 77%, 88%, 90%, 91%, and 93%, respectively, as stated in Table 4. Accordingly, MGA [19] is the weakest rejection approach and is unable to increase KNN accuracy, while EODA [23] is the second-best approach after CORT, able to increase KNN accuracy to 93%, which is greater than the 77% obtained by MGA [19] but less than the 95% reached by CORT.

As seen in Table 4, CORT had the lowest error value, at 5%, whereas MGA [19] had the highest, at 23%. Actually, the error of EODA [23] was 7%, which is more than the 5% attained by CORT but less than the 23% achieved by MGA [19]. Consequently, after CORT, EODA [23] is the second-ranked approach. The third-ranked approach based on error values is AAIC [22], which had a value of 9%.

MGA [19], OCFS [20], ROSIE [21], AAIC [22], EODA [23], and CORT had precision values of 76.4%, 87.5%, 89.6%, 90.3%, 92.6%, and 94.4%, respectively, as shown in Table 4. Furthermore, as shown in Table 4, the recall of MGA [19], OCFS [20], ROSIE [21], AAIC [22], EODA [23], and CORT was 76%, 87.3%, 89.4%, 90%, 92.2%, and 94.2%, respectively. Thus, CORT obtained the highest precision and recall values, whereas MGA [19] obtained the lowest. As indicated by the run times shown in Table 4, EODA [23] took the least time, while MGA [19] took the most. The run times for MGA [19], OCFS [20], ROSIE [21], AAIC [22], EODA [23], and CORT were 14, 12, 9, 7.3, 6.7, and 5 s.

This discussion indicates that CORT outshines other contemporary methods by presenting superior results. According to these findings, BCD is a quick, reliable, precise, efficacy, and adaptable technique, but it is also sophisticated, operates on a small dataset, and does not use multi-label data, as shown in Table 6.

According to the second scenario, BCD outperforms other techniques in [13,14,15,16,17,18]. According to Table 5, the BCD strategy presented superior accuracy, precision, recall, and F1-measure values while having the lowest error and shortest execution time.

In contrast, the technique in [13] had the lowest accuracy, precision, recall, and F1-measure scores, as well as the highest error and longest execution time. After BCD, the model in [18] is ranked as the second-best model, and the model in [17] ranks third. The proposed BCD strategy achieved 98.7% accuracy, 1.3% error, 98.4% precision, 98% recall, and an execution time of 3 s.

The model in [13] provided an accuracy of 80%, an error of 20%, a precision of 79.6%, a recall of 79.3%, an F1-measure of 79.449%, and a run time of 12 s. Furthermore, the model in [18] achieved an accuracy of 98%, an error of 2%, a precision of 97.4%, a recall of 97.2%, an F1-measure of 97.29%, and a run time of 4.3 s. Finally, the method in [17] obtained an accuracy of 97.7%, an error of 2.3%, a precision of 96.6%, a recall of 96.3%, an F1-measure of 96.44%, and a run time of 5.4 s.

This comparison shows that the suggested BCD strategy performs better than other strategies, providing a diagnosis quickly and precisely. Consequently, the BCD strategy is a rapid, reliable, accurate, efficacious, and scalable technique, but it remains difficult, deals with a small dataset, and does not use multi-label data as depicted in Table 6.

## 6. Conclusions and Future Works

Breast Cancer Discovery (BCD) is a revolutionary strategy for diagnosing breast cancer in women. BCD comprises two steps: The Pre-processing Step (P^2^S) and the Breast Cancer Discovery Step (BCDS). The filtered data from the P^2^S are forwarded to properly train the ECM in the BCDS by employing the IG method to select an optimal subset of features based on the WBCD dataset. Outliers are eliminated via the proposed CORT.

CORT consists of two phases: the quick rejection phase (QRP) and the accurate rejection phase (ARP). While the QRP may swiftly eliminate outliers utilizing standard deviation as a statistical method, the ARP may discard outliers precisely by deploying Binary Harris Hawk Optimization (BHHO). According to the experiments, the proposed BCD provides a faster and more accurate diagnosis than other techniques. The BCD strategy’s outcomes were 98.7%, 98.4%, 98%, 98.19%, 1.3%, and 3 s for accuracy, precision, recall, F1-measure, error, and run time, respectively.

In the future, the proposed BCD method will be tested on a variety of datasets of varying sizes. Incorporating alternative heuristic methods to identify the most significant features in the breast cancer dataset will enhance the BCD technique. This framework can be used to detect additional disorders, including chronic liver disease, Alzheimer’s disease, many types of cancer, and diabetes mellitus. To further improve detection accuracy, the BCD strategy will utilize additional classifiers in the BCDS.

## Figures and Tables

**Table 1:** Comparative review of various breast cancer discovery techniques.

Strategy	Description	Benefits	Drawbacks
[13] 2019	A mixed architecture that combines fuzzy systems, neural networks, and pruning technique concepts was presented. The goal of this work was to develop an integrated approach that can forecast breast cancer with high accuracy while also producing a specialized system that can identify sick individuals by using fuzzy rules to pull knowledge from the database.	Due to its one-step nature, the pruning approach performed significantly faster than the resampling method. This improved the model’s compactness and run time. In comparison to other recent investigations, there were notable improvements in accuracy, sensitivity, and specificity.	Using an integrated method might be computationally demanding, especially when working with very large datasets.
[14] 2023	The proposed discrete learning classification system has the potential to significantly improve medical decision-making accuracy compared to conventional direction-based continuous techniques.	The suggested model’s accuracy is a key advantage over other classifiers.	Limited capacity for interpretation. Considerable cost and processing time. The proposed model is complex to use and implement.
[15] 2023	This research highlighted the potential of machine learning to detect and diagnose breast cancer early, leading to better results for patients and healthcare.	The Random Forest model’s excellent accuracy implies its appropriateness for inclusion in clinical practice as an extra method to aid in breast cancer diagnosis.	The intrinsic difficulty of machine learning models may make it difficult to interpret their predictions. The investigation focused on the Wisconsin breast cancer diagnostic dataset, which may restrict its applicability to various datasets.
[16] 2024	This work presented a method to identify breast cancer instances as benign or malignant by leveraging machine learning techniques and 1D CNN feature extraction in order to improve accuracy.	The suggested method achieved the greatest accuracy (98.24%) in the detection and prognosis of breast cancer.	Considerable cost and processing time.
[17] 2023	Breast cancer detection uses machine learning algorithms including Support Vector Machine, Naïve Bayes, K-Nearest Neighbors, and AdaBoost. This study included eleven machine learning classifiers to find the most appropriate technique for breast cancer prediction.	A real-time collaborative web application was designed to detect breast cancer. The application in this work achieved accuracy of 98.77%. This study utilized eleven classifiers and various machine learning techniques, such as feature optimization methods including PCA and feature scaling, hyperparameter tuning, and cross-validation.	Computationally demanding. Considerable cost and processing time.
[18] 2023	The goal of this research was to enable radiologists to utilize machine learning and soft computing techniques to more rapidly and reliably identify and categorize breast cancer.	It selects the optimal feature subset by considering all of the input feature dimensions and developing an effective feature selection method. It tended to correctly recognize breast cancer with the highest degree of accuracy (98.9%) and the lowest cost of error.	Cannot manage large datasets. Adoption of online feature selection algorithms presents challenges because real-time data are required.

**Table 2:** Recent approaches to outlier rejection used in assessment strategies.

Outlier Detection Method	Explanation
MGA [19] 2019	This study proposed a set of objectives that make it possible to efficiently identify outliers with the use of a multi-objective genetic algorithm.
OCFS [20] 2021	This study investigated the effects of outliers and feature elimination on the Wisconsin Diagnostic Breast Cancer Dataset, which was investigated via seven distinct algorithms for machine learning in an effort to tackle these problems. The findings demonstrated that the classifiers using AdaBoost, Random Forest, and Logistic Regression eliminated outliers from the dataset with the highest accuracy.
ROSIE [21] 2022	This study suggested robust sparse ensemble for outlier detection (ROSIE), an ensemble classification technique that incorporates three basic and resilient algorithms for outlier detection and feature selection. It also includes a bootstrap-based validity test. ROSIE was used to identify outliers via the rank product test, which considered outlier ranks from all three approaches. The significant characteristics were those that were consistently selected by each technique.
AAIC [22] 2023	The detection of breast cancer using gene expression data was made possible by a combination approach that blends the CNN design with the Ebola optimization technique. Array–Array Intensity Correlation was one of the pre-processing techniques used to exclude outliers.
EODA [23] 2018	The feasibility of outlier identification was demonstrated using ensemble hypotheses based on genetic expression and clinical parameters for outlier patients.

**Table 3:** Confusion matrix equations.

Measure	Equation	Description
Precision (P)	TP / (TP+FP)	The proportion of correct positive predictions.
Recall (R)	TP / (TP+FN)	The proportion of instances with positive labels that were also projected to be positive.
Accuracy (A)	(TP+TN) / (TP+TN+FP+FN)	The proportion of predictions that are correct.
Error (E)	1−Accuracy	The proportion of predictions that are incorrect.
F1-measure	2∗P∗R/(P+R)	The weighted harmonic mean of precision and recall.

**Table 4:** Comparison between CORT and other recent outlier rejection techniques.

Technique	Accuracy (%)	Precision (%)	Recall (%)	Error (%)	F1-Measuer (%)	Run Time (s)
MGA [19]	77	76.4	76	23	76.199	14
OCFS [20]	88	87.5	87.3	12	87.399	12
ROSIE [21]	90	89.6	89.4	10	89.499	9
AAIC [22]	91	90.3	90	9	90.149	7.3
EODA [23]	93	92.6	92.4	7	92.499	6.7
**Proposed CORT**	**95**	**94.4**	**94.2**	**5**	**94.299**	**5**

**Table 5:** Comparison between BCD and other recent detection strategies.

Technique	Accuracy (%)	Precision (%)	Recall (%)	Error (%)	F1-Measure (%)	Run Time (s)
[13]	80	79.6	79.3	20	79.449	12
[14]	93	92.5	92.4	7	92.449	9
[15]	95.5	94.2	94.1	4.5	94.149	7.8
[16]	97	96.1	95.8	3	95.949	6
[17]	97.7	96.6	96.3	2.3	96.449	5.4
[18]	98	97.4	97.2	2	97.299	4.3
**Proposed BCD strategy**	**98.7**	**98.4**	**98**	**1.3**	**98.199**	**3**

**Table 6:** The benefits and drawbacks of the proposed BCD strategy.

Benefits		Drawbacks	
Characteristic	Overview	Characteristic	Overview
Reliability	BCD is a reliable strategy since it can determine patients considering a variety of nuclear features.	Size of dataset	Although BCD uses the 699 examples from the WBCD dataset to illustrate different contemporary strategies, additional cases should be evaluated in order to yield more precise conclusions.
Quickness	BCD is a rapid deterministic strategy because an ECM can make choices based on accurate data that are free of outliers.	Complexity	BCD is a complex strategy because it comprises two layers, each containing several steps that are implemented consecutively.
Number of features	BCD has the capacity to handle a very large number of features and choose the optimal subset of them before using an ECM to recognize patients with certainty.	Multi-label classification	BCD is an assessment strategy that uses binary classification to divide instances into benign and malignant classes. As a result, multi-label data in the dataset should be used to test BCD.
Efficacy	BCD is a useful strategy because it can make quick, precise determinations.		
Accuracy and precision	BCD can precisely diagnose breast cancer patients by utilizing an ECM after removing outliers and superfluous information from the data.		

## Data Availability

https://archive.ics.uci.edu/dataset/15/breast+cancer+wisconsin+original (accessed on 1 July 2024).
